# 

**Published:** 1859-08

**Authors:** 


### *New Publications Received.*

Anatomy, Descriptive and Surgical. By Henry Gray, F.R.S., Lecturer on Anatomy at St. George's Hospital. Blanchard & Lea, Philadelphia. From the publishers.

On Poisons, in relation to Medical Jurisprudence and Medicine. By Alfred Swaine Taylor, M.D., F.R.S., etc. Second American from the second and revised London edition. Blanchard & Lea, Philadelphia. From the publishers.

A Manual of Elementary Chemistry, Theoretical and Practical. By George Fownes, F.R.S., etc. From the seventh revised and corrected London edition. Edited by Robert Bridges, M.D. Blanchard & Lea, Philadelphia. From the publishers.

Urinary Deposits: Their Diagnosis, Pathology, and Therapeutical Indications. By Golding Bird, M.D. Edited by Edmund Lloyd Birkett, M.D., etc. A new American from the fifth London edition. Blanchard & Lea, Philadelphia. From the publishers.

A Practical Treatise on the Diseases of Infancy and Childhood. By T. H. Tanner, M.D., F.L.S., etc. Lindsay & Blakiston, Philadelphia. From the publishers.

Five Essays. By John Kearsley Mitchell, M.D., late Professor of Practice of Medicine in Jefferson College, etc. Edited by S. Wier Mitchell, M.D. J. B. Lippincott & Co., Philadelphia. From the publishers.

Green on Bronchitis. Fourth edition, revised and enlarged. Wiley & Halsted, N. Y. From the publishers.

Contributions to Midwifery, and Diseases of Women and Children, with a report on the progress of Obstetrics and Uterine and Infantile Pathology, in 1858. By E. Noeggreath, M.D., and A. Jacobi, M.D. Baillière Brothers, N. Y. From the authors.

A Treatise on Gonorrhea and Syphilis. By Silas Durkee, M.D., etc. John P. Jewett & Co., Boston. From the author.

Transactions of the New Jersey State Medical Society, for 1859.

Introductory Address, delivered at the opening of the Medical Department of the University of the Pacific, at San Francisco, California, May 13th, 1859, and addresses by Rev. Dr. Peck, and Rev. Mr. Cutler.

The Brooklyn City Hospital in 1858, and the address of J. G. Hutchinson, M.D., one of the attending Surgeons.

An Address on the Registration of Disease. Printed with additions, from the N. Y. State Medical Society, 1859. By Wm. C. Rogers, M.D.

A Lecture on Idiocy. By John M. Galt, M.D., Superintendent and Physician of the Eastern Lunatic Asylum of Virginia, at Williamsburg.

Transactions of the N. Y. Academy of Medicine. Anatomy of the Placenta. Physical and Chemical changes in the Interior of the Body. By John C. Dalton, Jr., M.D.

The above publications will receive early notice.

